# CTX-M-15–producing Enteroaggregative *Escherichia coli* as Cause of Travelers’ Diarrhea

**DOI:** 10.3201/eid1710.110022

**Published:** 2011-10

**Authors:** Elisabet Guiral, Eva Mendez-Arancibia, Sara M. Soto, Pilar Salvador, Anna Fàbrega, Joaquim Gascón, Jordi Vila

**Affiliations:** August Pi i Sunyer Biomedical Research Institute, Barcelona, Spain (E. Guiral, E. Mendez-Arancibia, S.M. Soto, P. Salvador, A. Fàbrega, J. Vila);; Barcelona Centre for International Health Research, Barcelona (J. Gascón);; University of Barcelona, Barcelona (J. Vila)

**Keywords:** bla_CTX-M-15_, enteroaggregative Escherichia coli, diarrhea, resistance mechanisms, travelers’ diarrhea, bacteria, dispatch

## Abstract

Travelers’ diarrhea is a major public health problem. From patients in whom diarrhea developed after travel to India, 5 enteroaggregative *Escherichia coli* strains carrying β-lactamase CTX-M-15 were identified; 3 belonged to clonal complex sequence type 38. This β-lactamase contributes to the multidrug resistance of enteroaggregative *E. coli*, thereby limiting therapeutic alternatives.

Travelers’ diarrhea remains a major public health problem, causing substantial illness and disability. Almost 50% of patients with travelers’ diarrhea require treatment with antimicrobial drugs because of persistence or severity of signs and symptoms (*1*). Enteroaggregative *E. coli* (EAEC) is among the most common diarrheagenic *E. coli* pathotypes recognized (*2*). The first-choice agents for treating EAEC infections are quinolones, rifaximin, azithromycin, and cephalosporins. However, the number of pathogenic *E.* *coli* strains resistant to multiple antimicrobial agents has increased, and resistance to third-generation cephalosporins (e.g., ceftazidime, ceftriaxone, or cefotaxime) associated with production of extended-spectrum β-lactamases (ESBLs) limits therapeutic options (*3*).

Although ESBL production has mainly been shown in extraintestinal *E. coli* infections, studies concerning effects of ESBLs in intestinal *E. coli* infections are scarce. The worldwide spread of CTX-M-15 type ESBLs has led these β-lactamases to replace TEM- and SHV-type ESBLs in Europe, Canada, and Asia and become one of the major groups of ESBLs studied. Of the different CTX-M–type ESBLs, CTX-M-15 has become the most widely distributed enzyme worldwide. It was first identified in an isolate from India in 1999 and thereafter became prevalent around the world (*4*). CTX-M-15 enhances hydrolytic activity against ceftazidime (*5*). A particular clone of CTX-M-15–producing *E. coli*, characterized by phylogenetic type (phylotype) B2 and sequence type 131 (ST131), seems to be largely responsible for international epidemics of CTX-M–producing *E. coli* (*6*). Sequence types (STs) are grouped into clonal complexes by their similarity to a central allelic profile.

ST131 is a singleton and therefore does not belong to a clonal complex (*7*). Molecular epidemiologic studies have suggested that the sudden increase in CTX-M-15–producing *E. coli* worldwide was mainly caused by this single clone (ST131) and that foreign travel to high-risk areas, such as the Indian subcontinent, might play a partial role in the spread of this clone across continents (*8*). The *bla*_CTX-M-15_ gene is usually found downstream from the insertion sequence IS*Ecp1*, which may be involved in the clone’s dissemination and expression (*9*). We describe molecular epidemiology and plasmid analyses of 5 CTX-M-15–producing EAEC isolates from patients with travelers’ diarrhea who had traveled from Spain to India.

## The Study

The study included all patients with diarrhea who visited the Tropical Medicine Unit of Hospital Clinic in Barcelona, Spain, during 2005 and 2006. Patients with diarrhea that started during or shortly after (<5 days) a stay in a developing country were eligible. After the participants provided informed consent, clinical and epidemiologic data were collected.

Among all eligible participants, infection with EAEC and no other enteropathogen was found for 51. Of these 51 EAEC isolates, 5 from patients who had traveled to India were resistant to third-generation cephalosporins. Resistance phenotypes indicated ESBL production. MICs for antimicrobial agents and susceptibility class were determined by using the Clinical and Laboratory Standards Institute breakpoints guideline (Table 1). All strains were resistant to penicillins; second-, third-, and fourth-generation cephalosporins; and all β-lactamase–inhibitor combinations except piperacillin/tazobactam. Apart from β-lactam susceptibility, the strains showed resistance to other classes of antimicrobial agents, such as fluoroquinolones, tetracyclines, and monobactams (aztreonam). Positive amplification with specific primers and sequencing for the *bla*_CTX-M-15_ gene provided positive genotypic confirmatory test results for ESBL production.

**Table 1:** Susceptibility of 5 enteroaggregative *Escherichia.coli* strains that produced diarrhea in patients returning from India, 2005–2006*

Strain	Antimicrobial agent																							
	AM	PR	AG	P/T	A/S	FU	FOX	FZ	PIM	CTX	CAZ	GN	AK	TB	F	IMI	ME	AZ	CIP	NOR	LEV	TE	SXT	CL
HC19	R	R	R	S	R	R	I	R	R	R	R	R	S	R	S	S	S	R	R	R	R	S	R	S
HC64	R	R	R	S	R	R	I	R	R	R	R	S	S	R	S	S	S	R	R	R	R	R	R	S
HC67	R	R	R	S	R	R	I	R	R	R	I	R	R	R	S	S	S	R	R	R	R	R	R	S
HC74	R	R	R	S	R	R	I	R	R	R	R	S	R	R	S	S	S	R	R	R	R	R	R	R
HC76	R	R	I	S	R	R	S	R	I	R	S	S	S	S	S	S	S	I	R	R	R	R	R	S

*AM, ampicillin; PR, piperacillin; AG, amoxiclavulanic acid/augmentin; P/T, piperacillin/tazobactam; A/S, ampicillin/sulbactam; FU, cefuroxime; FOX, cefoxitin; FZ, cefazoline; PIM, cefepime; CTX, cefotaxime; CAZ, ceftazidime; GN, gentamicin; AK, amikacin; TB, tobramycin; F, fosfomycin; IMI, imipenem; ME, meropenem; AZ, aztreonam; CIP, ciprofloxacin; NOR, norfloxacin; LEV, levofloxacin; TE, tetracycline; SXT, cotrimoxazole; CL, chloramphenicol; R, resistant; S, sensitive; I, intermediate.

**Figure 1:**
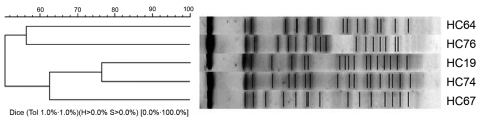
Cluster analysis of the enteroaggregative *Escherichia coli* strains from the pulsed-field gel electrophoresis fingerprinting.

The epidemiologic relationships among the 5 strains were studied by repetitive sequence–based PCR, pulsed-field gel electrophoresis, and multilocus sequence typing (*10**,**11*). The PCR and pulsed-field gel electrophoresis genomic fingerprinting showed that the 5 strains were not epidemiologically related (Figure 1). However, multilocus sequence typing identified 2 clonal complexes: ST38 (3 strains) and ST10 (1 strain). The fifth strain could not be classified into any clonal complex (Table 2).

**Table 2:** Analysis results for 5 enteroaggregative *Escherichia coli* strains that produced travelers’ diarrhea in patients returning from India, 2005–2006*

Strain	PFGE type	MLST clonal complex	Phylotype	Genes encoding for virulence factors	*bla*_CTX-M-15_ location
HC19	A	ST38	D	*aat, aap, aggR, aggA*	Chromosome
HC64	B	None	B2	*aat, astA,sat*	Plasmid
HC67	C	ST38	D	*aat, astA*	Plasmid
HC74	A_1_	ST38	D	*aat, aap, aggR, aggA, afn43, fyuA*	Chromosome
HC76	D	ST10	B2	*aat, fyuA*	Plasmid

*PFGE, pulsed-field gel electrophoresis; MLST, multilocus sequence type; ST, sequence type.

*E. coli* strains were classified into phylogenetic groups by multiplex PCR, described by Clermont et al. (*12*). The 3 strains in clonal complex ST38 belonged to the potentially virulent phylogenetic group D; the other 2 belonged to group B2 (Table 2).

A PCR method was used to detect genes encoding for typical EAEC virulence factors (*2*). These genes include *aggA* and *aafA* (encoding for adhesions); *aap* (for dispersin); *aatA* (for TolC); *aggR* (for regulation of aggregation); *astA*, *set1A*, and *sen* (for toxins), *fyuA* (for iron recruitment); *agn43* (for antigen 43); and genes encoding for serine protease autotransporter toxins such as *pet* and *sat*. Gene *aatA* was detected in the 5 strains, whereas *aap*, *aggR*, and *aggA* had positive amplification for only 2 of the strains belonging to ST38. The other genes detected are shown in Table 2. EAEC was also identified by typical adherence to HEp-2 cells.

To determine the genetic environment of the *bla*_CTX-M-15_ gene, we designed an inverse PCR. We designed the primers by studying the gene sequence and were directed outside the gene. The IS*Ecp1* insertion sequence was upstream from the *bla*_CTX-M-15_ gene, which was also confirmed by PCR of the specific insertion sequence. To confirm the possible relationship between IS*Ecp1* and the resistance *bla*_CTX-M-15_ gene_,_ we conducted a PCR with the forward primer for the IS*Ecp1* and the reverse primer for the *bla*_CTX-M-15_ gene.

**Figure 2:**
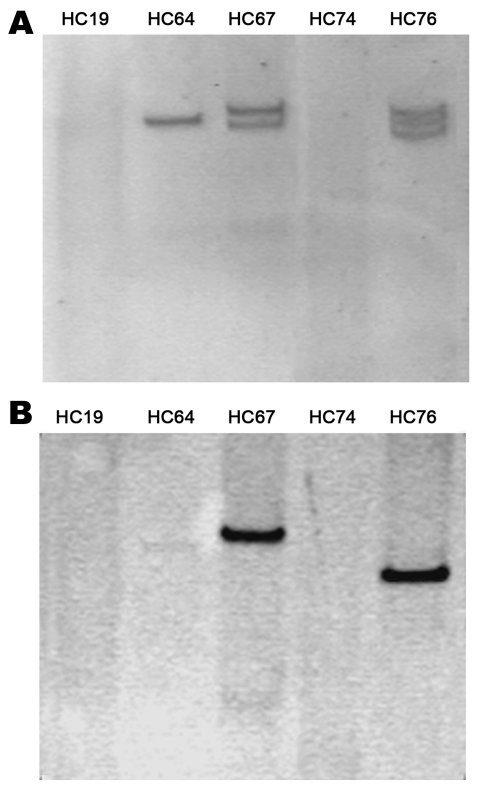
Plasmidic profile of the enteroaggregative *Escherichia coli* strains (A) and Southern blotting of the *bla*_CTX-M-15_ gene (B).

For plasmid extraction of the 5 isolates, we used the method of Kado and Liu (*13*). Only 3 strains had plasmids ranging from 93 kb to 170 kb (Figure 2, panel A). To confirm the absence of plasmids in the 2 strains, we conducted S1 digestion of the strains, resolving chromosomal DNA from plasmidic DNA. Southern blot of this digestion showed that the *bla*_CTX-M-15_ gene was chromosomally located in these 2 strains, as was the *aatA* gene (usually found in the plasmid contained in EAEC strains) (data not shown). Finally, the location of the *bla*_CTX-M-15_ gene in the 3 plasmid-containing strains was analyzed by using Southern blot from the plasmid extraction. The *bla*_CTX-M-15_ gene was located in a plasmid in the 3 strains. The size of the plasmid containing CTX-M-15 varied in each strain (Figure 2, panel B). Plasmids with specific known molecular weight were used to provide a range of the size of the plasmids studied.

## Conclusions

We identified several features concerning the molecular epidemiology of CTX-M-15–producing EAEC isolates in India. First, all strains belonged to phylogenetic groups D and B2, the 2 groups most commonly found with *E. coli* infections (*14*). Second, not finding ST131 suggests that ST131 might not be the most common ST among EAEC strains from India and that clonal complex ST38 might play a large role in causing infectious intestinal diseases. Third, the *bla*_CTX-M-15_ gene is not only located in the plasmid but may also be in the chromosome. However, previous reports have shown that *bla*_CTX-M-15_ is consistently linked with IS*Ecp1*, which means that the chromosomal location might have originated from a previous plasmid location that was part of either a transposon or a cassette within an integron (*9*). It is also worth noting that the size of the plasmids containing the *bla*_CTX-M-15_ gene was not the same in all strains, indicating that this gene may be located in different types of plasmids.

This evidence of widespread distribution and flexibility of the *bla*_CTX-M-15_ gene highlights the need to develop appropriate means to control dissemination of this gene and associated resistance genes. Epidemiologic surveillance and correct use of antimicrobial agents will help prevent the steady increase of antimicrobial drug resistance worldwide.
